# The Physical and Mental Metaphors (PMM) task and its validation in schizophrenia: A tool for rapid assessment of figurative language comprehension, between pragmatics and symptoms^☆^

**DOI:** 10.1016/j.scog.2026.100448

**Published:** 2026-07-01

**Authors:** Luca Bischetti, Valentina Bambini, Giulia Agostoni, Margherita Bechi, Mariachiara Buonocore, Jacopo Sapienza, Federico Frau, Ginevra Martinelli, Chiara Pompei, Biagio Scalingi, Marco Spangaro, Francesca Martini, Federica Cocchi, Roberto Cavallaro, Marta Bosia

**Affiliations:** a Laboratory of Neurolinguistics and Experimental Pragmatics (NEPLab), Department of Humanities and Life Sciences, University School for Advanced Studies IUSS, Pavia, Italy; b Schizophrenia Research and Clinical Unit, IRCCS San Raffaele Scientific Institute, Milan, Italy; c School of Medicine, Vita-Salute San Raffaele University, Milan, Italy

**Keywords:** Physical and Mental Metaphors (PMM) task, APACS, Metaphor, Pragmatics, Disorganization, Psychosis, Formal Thought Disorder, Concretism

## Abstract

Difficulties in figurative language understanding are largely documented as part of a broader pragmatic impairment, especially in psychosis. Among figurative domains, metaphor stands out as a significant predictor of functioning, in addition to exhibiting meaningful associations with psychopathological and cognitive features. Yet, the assessment of metaphor comprehension remains a niche area, also due to the lack of reliable and valid tools. Here, we examined the psychometric properties of a rapid test of metaphor comprehension, the Physical and Mental Metaphors (PMM) task, involving a sample of 143 patients with schizophrenia and 57 controls.

Findings provide robust support for the PMM reliability and validity. First, internal consistency was satisfactory, and the factorial structure aligned with theoretical assumptions. Second, the PMM demonstrated good validity, being strongly correlated with the pragmatic profile of patients as measured with a standard tool such as the Assessment of Pragmatic Abilities and Cognitive Substrates (APACS) test, showing also relevant associations with symptomatology, in particular abstract thinking and the broader domains of disorganization and negative symptoms, as well as weakly with Theory of Mind and global cognition. Third, the PMM task proved highly effective both in discriminating between individuals with and without pragmatic deficit, as detected via APACS, and in distinguishing patients from healthy controls.

Taken together, these results indicate that the PMM task captures the specific impairment in figurative language and the general pragmatic vulnerability characteristic of schizophrenia, as well as their interface with symptoms. As such, it represents a valuable tool for detecting communicative disorders, characterizing symptoms and complementing the diagnostic process.

## 1 Introduction

Pragmatic abilities, defined as the capacity to integrate contextual factors with linguistic meaning in order to interpret utterances, including inferring the speaker's intended meaning and producing contextually appropriate and adequately informative contributions to discourse (Bosia et al., 2016; Levinson, 1983; Sperber and Wilson, 1995), are the pinnacle of human verbal capacity and crucially serve social functioning (Cummings, 2016; Snow and Douglas, 2017). At the same time, these abilities are extremely vulnerable across a range of clinical conditions, from developmental disorders, such as autism, to acquired impairments in adulthood (Alduais et al., 2022; Bischetti et al., 2024a). This vulnerability is particularly evident in psychosis, where pragmatic abilities are severely compromised (Cayouette et al., 2025; Meister et al., 2026; Perlini et al., 2018; Tavano et al., 2008), to the point that, in schizophrenia, the pragmatic deficit constitutes a core feature (Bambini et al., 2016) linked to specific neurophysiological alterations (Agostoni et al., 2026; Kuperberg et al., 2010, Kuperberg et al., 2018). Importantly, studies have documented a profound impact of pragmatic difficulties on daily living, particularly on social relationships (Agostoni et al., 2021), pointing to the importance of considering this dimension in routine clinical assessment.

Among the most impaired aspects of pragmatics in psychosis is figurative language comprehension (Bambini et al., 2016; Perlini et al., 2018), namely the comprehension of non-literal expressions such as proverbs (e.g., Opportunity makes the thief), metaphors (e.g., That lawyer is a shark), and idioms (e.g., Take the bull by the horns). Studies reported impairment in figurative language as diffuse as around 80% in schizophrenia (Bambini et al., 2016), with difficulties being present in psychosis in general (Perlini et al., 2018), from major depressive to bipolar disorders (Mueller et al., 2025; Nagels et al., 2016). These difficulties are long known in psychopathology, where they are traditionally labelled under the term concretism (Bleuler, 1911; Harrow et al., 1974), defined as an impairment in abstract thinking that restricts the individual to the immediate, concrete features of a stimulus or situation, resulting in a tendency to interpret figurative expressions in a literal rather than non-literal way (Bambini et al., 2025).

The evaluation of concretism is indeed embedded in specialized tools for clinical assessment, especially of negative symptoms, such as the Positive and Negative Syndrome Scale (PANSS; Kay et al., 1987), and Formal Thought Disorder, such as the Thought and Language Disorder (TALD; Kircher et al., 2014). Yet, the assessment of figurative language comprehension as done in these clinical scales is often coarse-grained from the linguistic point of view, and limited to proverbs. Linguistically oriented studies showed that idioms, metaphors, and proverbs capture different nuances of concretism. For instance, proverbs are the most impaired type of figurative language across different testing formats, possibly due to their content highly relying on social wisdom, with also a close connection with cognitive and socio-cognitive skills (Brüne, 2003, Brüne, 2005), while idioms seem to be more easily understood, possibly because of their conventionality (Rapp et al., 2018). As for metaphors, their impairment, rooted in brain functioning alterations (Adamczyk et al., 2021; Kircher et al., 2007), turned out to be particularly well suited to capture functional outcome (Adamczyk et al., 2016; Rossetti et al., 2018), possibly because it is not as widespread as in the case of proverbs and hence it detects more subtle variations in daily functioning. Moreover, metaphor comprehension deficits have also been linked to the severity of specific symptoms (Karabanowicz et al., 2020; Siddi et al., 2016).

Given the importance of metaphor as part of the pragmatic profile and for functional outcome, we decided to validate a tool specifically devoted to the assessment of metaphor comprehension, filling a current gap in the literature. Here, we introduce a brief test of metaphor comprehension, the Physical and Mental Metaphors (PMM) task, grounded in linguistic theories of metaphor comprehension and including items with different levels of complexity. Originally developed to track the maturation of metaphor abilities throughout childhood (Lecce et al., 2019; Pompei et al., 2025; Tonini et al., 2023), the PMM task was constructed to assess different facets of metaphor comprehension, intended as a multimodal process that might capitalize on different verbal, cognitive and sensory-motor experience (Battaglini et al., 2025; Winner et al., 1976). Specifically, the PMM task includes 14 metaphors, half referring to physical/behavioral characteristics (e.g., “Some singers are nightingales”, meaning that they sing very well) and thus capitalizing on perceptual experience, half referring to mental characteristics (e.g., “Some friends are anchors”, meaning that they are supportive and you can count on them) and thus capitalizing on social cognition skills, presented in an open response format asking participants to provide a verbal explanation. Building on this, in the present work, we aim to validate the use of the PMM task in schizophrenia by assessing its reliability and validity against established pragmatic assessment measures, alongside its relationship with symptomatology, Theory of Mind, and cognition. Moreover, we evaluated its ability to capture pragmatic impairment in patients and to establish its potential utility in distinguishing affected individuals from healthy controls.

## 2 Methods

### 2.1 Participants

For this project, we pooled data from four independent studies, yielding a total sample of 200 participants. Participants with schizophrenia were drawn from the *Pragmatics of Communication* study (PRAGMACOM; Bambini et al., 2022) and *The Fragility of Metaphors* (*FraMe*)*: Learning, Losing, and How to Train Them* project (FRAME; ClinicalTrials.gov ID: NCT06375902), based on the availability of PMM assessment data. Overall, 143 participants with schizophrenia were included. Additional measures were available for subsets of this group, including PANSS (*n* = 121), BACS (*n* = 120), APACS (*n* = 58), and PST (n = 58). PMM retest data were available for 14 patients.

Healthy control participants were selected from Agostoni et al. (2024) and Ceccato et al. (2025) based on the availability of PMM data, yielding a total of 57 participants. For this group, APACS, PST, and BACS data were available for 34 individuals.

All participants were native Italian speakers and had no concomitant major neurological conditions. Enrollment procedures and study protocols are described in detail in the respective original publications (see references above).

### 2.2 Assessment

#### 2.2.1 Physical and Mental Metaphors (PMM) Task

**Fig. 1:**
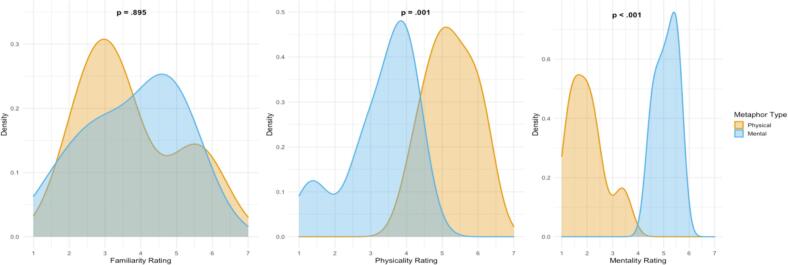
Density plots illustrating group differences in familiarity, physicality, and mentality, respectively, with statistical comparisons based on independent-samples *t*-tests. Physical metaphors are shown in orange, while mental ones are shown in blue.

The Physical and Mental Metaphors (PMM) task is a measure of metaphor comprehension originally developed for middle childhood (Del Sette et al., 2020; Lecce et al., 2019; Tonini et al., 2023) and later adapted for adults (Bambini et al., 2020c; Ceccato et al., 2025). The task includes 14 non-conventional metaphors of varying complexity, operationalized in two distinct sets that require either inferences about physical/behavioral attributes (physical metaphors; e.g., “Some singers are nightingales”) or about more complex mental states (mental metaphors; e.g., “Some friends are anchors”). Structurally, each expression is a nominal predicative metaphor in the form *X*s are *Y*s, with *X*s (topics) being common nouns denoting human beings and *Y*s (vehicles) being common nouns denoting concrete non-human entities. Familiarity values, physicality (i.e., how much each metaphor describes the subject in terms of physical or behavioral qualities) and mentality (i.e., how much each metaphor describes the subject in terms of psychological attributes, including personality or mental content, such as emotions, thoughts, and desires) were collected on 1–7 Likert-type scales in previous studies (Canal et al., 2022; Bressler et al., 2026). Their mean values, for the whole PMM task and separately for each set, are reported in Table 1 and plotted in Fig. 1. Mean comparisons confirmed that metaphors were of similar, medium familiarity across sets and that metaphorical relationships between topics and vehicles differed between sets. The complete list of items, along with their psycholinguistic features, can be found in the Zenodo repository (https://doi.org/10.5281/zenodo.18120067).

**Table 1:** Linguistic properties of items in the PMM task.

		Physical and Mental Metaphors task	Physical metaphors	Mental metaphors	*p*-value (from independent-samples *t*-tests)
Familiarity	Mean (SD)	3.74 (1.32)	3.69 (1.38)	3.79 (1.36)	*t*(12) = −0.13, *p* = .895
	Range	1.64–5.88	1.64–5.46	2.88–5.88	–
Physicality	Mean (SD)	4.26 (1.31)	5.24 (0.69)	3.28 (1.00)	*t*(12) = 4.24, *p* = .001
	Range	1.36–6.14	4.21–6.14	1.36–4.25	–
Mentality	Mean (SD)	3.56 (1.71)	2.01 (0.74)	5.11 (0.43)	*t*(12) = −9.56, *p* < .001
	Range	1.25–5.57	1.25–3.39	4.50–5.57	–

The main outcome of the PMM task is a measure of accuracy, defined as the ability to understand and articulate the link between the topic and the vehicle of the metaphor (see Ceccato et al., 2025, and Del Sette et al., 2020 for further details). For each item, possible scores are: 0 for incorrect, don't know, and literal responses; 1 for partially correct responses and responses focusing on non-salient features of the vehicle; and 2 for fully correct and salient responses. Scores are summarized as Total Accuracy (range 0–28), Physical Accuracy (range 0–14), and Mental Accuracy (range 0–14) scores, which reflect the participant's ability to correctly interpret metaphorical meanings (overall and for each set). On the pooled data included here, a second judge (LB, a linguist) independently scored metaphor accuracy for 60 patients (∼40% of the total sample) for the purpose of assessing inter-rater reliability of the PMM relative to the original scores assigned by a clinician.

In addition, the PMM task also evaluates the type of interpretation on a scale from physical to psychological, based on the use of words reflecting emotions, thoughts, or desires (see Ceccato et al., 2025). For the purpose of this study, we focused on accuracy scores. See the Zenodo repository for additional analysis on the interpretation score (https://doi.org/10.5281/zenodo.18120067).

The adult PMM task is available in three forms, with comparable mean familiarity ratings (form A, *M* = 3.74, *SD* = 1.32; form B, *M* = 3.74, *SD* = 1.31; form C, *M* = 3.79, *SD* = 1.20) and Cronbach's *α*s (main version, form A, *α* = 0.78, 95% CI [0.66, 0.89]; form B, *α* = 0.75, 95% CI [0.60, 0.89]; form C, *α* = 0.73, 95% CI [0.57, 0.89]), all strongly inter-correlated (*r*s ≥ 0.72) (see Bambini et al., 2022).

The average administration time of the PMM ranges between approximately 4 and 6 min, making the PMM suitable for routine clinical assessment.

For the purpose of this study, we focused on form A of the PMM task.

#### 2.2.2 Assessment of Pragmatic Abilities and Cognitive Substrates (APACS)

The Assessment of Pragmatic Abilities and Cognitive Substrates (APACS; Arcara and Bambini, 2016) is a validated test for evaluating expressive and receptive pragmatic skills across six tasks: Interview, Description, Narratives, Figurative Language 1, Humor, and Figurative Language 2. For the present study, the APACS Total score was used as a measure of overall pragmatic functioning.

#### 2.2.3 Clinical and cognitive measures

Additional measures included the Positive and Negative Syndrome Scale (PANSS; Kay et al., 1987), the Brief Assessment of Cognition in Schizophrenia (BACS) and the Picture Sequencing Task (PST).

As for the PANSS scale, consistent with Bambini et al., 2020a, Bambini et al., 2022, analyses focused on the three primary symptom scales: Positive, Negative, and General Psychopathology, as well as the specific subdomains N5 (Difficulty in Abstract Thinking) and P2 (Conceptual Disorganization). The Disorganization scale proposed by van der Gaag et al. (2006) was also computed to provide a detailed measure of thought and speech disorganization.

The BACS, Italian version (Anselmetti et al., 2008) is a validated neuropsychological tool for the assessment of cognition in schizophrenia. The analysis focused on the Cognitive Index (i.e., the mean of equivalent scores of each BACS subtest) as a summary measure. The PST (Brüne, 2003, Brüne, 2005) is a task using cartoon sequences to evaluate the ability to infer others' mental states. The analysis focused on the overall score of Theory of Mind abilities as a summary measure.

### 2.3 Statistical analysis

We verified comparability of data across samples of patients and control participants with Fligner-Killeen non-parametric tests and dispersion indices such as variance and SD ratios, as well as Median Absolute Deviation (MAD). Reliability analysis on the PMM task was conducted on the sample of patients with schizophrenia and healthy controls pooled together. Internal consistency was determined using Cronbach's α and McDonald's ω with 95% bootstrap confidence intervals (CIs). Item analyses were conducted within the framework of classical test theory, including examination of inter-item and item-total correlations and item difficulty indices. Dimensionality was confirmed with a factor analysis, informed by a parallel analysis to determine the number of factors, with principal axis factoring extraction and *promax* rotation (Revelle, 2025). Additionally, a two-parameter logistic (2PL) analysis (Kean et al., 2018) was conducted to account for differences in how effectively items discriminate along the latent trait continuum as a function of item difficulty. Moreover, a Wright map (Torres Irribarra and Freund, 2014) was created to visualize the alignment between item difficulty and respondents' ability levels within the full range of the latent trait.

Test-retest reliability was verified by correlating the performance in the PMM task in the sample of patients with two evaluations conducted with the form A of the test.

Inter-rater reliability was calculated as the correlation between two independent raters with the Intraclass Correlation Coefficient (ICC), selecting a two-way random-effect model testing agreement on the average score with the *irr* package (Gamer et al., 2019).

Validity analysis of the PMM task was conducted on the total sample. Construct and concurrent validity were calculated via Pearson's *r* correlations between PMM task scores and demographic, clinical, symptoms' severity, cognitive, and pragmatic measures, with Benjamini-Hochberg correction applied for multiple comparisons (FDR).

Discriminant validity was assessed in two steps. First, performance in the PMM task in patients and healthy controls was compared using independent-samples *t*-tests. Group discrimination of patients with schizophrenia vs. controls and of individuals with vs. without pragmatic deficits (assessed via the APACS test in a pooled sample of patients and control participants) was quantified with two distinct Receiver Operating Characteristic (ROC) curves, reporting the following measures: Area Under the Curve (AUC), sensitivity, specificity, and optimal thresholds maximizing the Youden's J statistic (Robin et al., 2011). AUCs are reported with 95% CIs and compared using DeLong's test (DeLong et al., 1988).

Analyses were conducted in R (R Core Team, 2023), v. 4.3.2.

## 3 Results

### 3.1 Sample data

The sample is described in Table 2.

**Table 2:** Demographic, clinical, and cognitive characteristics of patients with schizophrenia and controls.

	Patients with schizophrenia Mean (SD) [range] *n* = *143*	Controls Mean (SD) [range] *n* = *57*	*p*-value (from independent-samples *t*-tests)
Age	44.00 (13.49) [19–80]	47.97 (13.57) [20–63]	.063
Sex (M/F/NA)	87/54/2 (60.8/37.8/1.4%)	18/39 (31.6/68.4%)	–
Education (in years)	12.40 (2.92) [5–22]	11.88 (2.62) [8–21]	.240
Illness duration (in years)	19.91 (11.07) [0–55]	–	–
Pragmatics (APACS Total score) [score range, 0–1]	0.83 (0.09) [0.46–0.96]	0.95 (0.03) [0.83–0.99]	< .001
PANSS - Positive (Pos) Symptoms [score range, 7–49]	15.74 (4.47) [7–29]	–	–
PANSS P2 [score range, 1–7]	2.42 (1.26) [1–6]	–	–
PANSS - Negative (Neg) Symptoms [score range, 7–49]	20.52 (4.96) [9–35]	–	–
PANSS N5 [score range, 1–7]	3.23 (1.22) [1–7]	–	–
PANSS - General Psychopathology (Gen) [score range, 16–112]	39.12 (7.03) [20–62]	–	–
PANSS – Disorganization scale (Dys) [score range, 7–56]	20.73 (5.36) [8–35]	–	–
Cognition (BACS) [score range, 0–4]	1.71 (0.91) [0–3.33]	1.97 (0.81) [0.33–3.67]	.122
Theory of Mind (Picture Sequencing Task) [score range, 0–59]	48.33 (10.09) [13–59]	54.27 (5.10) [37–59]	< .001

**Note.** APACS and PST data were available for 58 patients with schizophrenia and 34 controls. BACS scores were available for 120 patients and 34 controls, and PANSS scores for 121 patients.

Fligner-Killeen tests supported the homogeneity of variance within clinical samples (all *p*s ≥ .058) and within healthy control samples (all *p*s ≥ .101), also confirmed by variance ratios (max/min < 2.50), SD ratios (all <1.58), and MAD ratios (all ≤2.00) within acceptable thresholds (Dean and Voss, 1999; Wilcox, 1987; Leys et al., 2013), thereby justifying the pooling of the samples into the two groups for subsequent analyses.

### 3.2 Performance in the PMM task

Scores obtained in the PMM task are reported in Table 3. Mean comparisons between groups showed that patients with schizophrenia scored significantly lower than controls in all indexes of the PMM, that is, total accuracy score (*t*(163.91) = −6.38, *p* < .001) and accuracy of physical (*t*(177.95) = −9.00, *p* < .001) and mental metaphors (*t*(152.55) = −3.18, *p* = .002). Between types (physical vs. mental), a significant difference was found in controls (Δ*M* = 1.49; *t*(56) = 5.03, *p* < .001), indicating that physical metaphors were easier than mental ones for the non-clinical group. In contrast, such difference was not found in patients with schizophrenia (Δ*M* = −0.08; *t*(142) = −0.27, *p* = .787).

**Table 3:** Performance of patients with schizophrenia and controls in the PMM task.

	Patients with schizophrenia	Controls	*p*-value (from independent-samples Welch's *t*-tests)
Accuracy - Total score [Possible range, 0–28]			
Mean (SD)	17.67 (6.16)	22.26 (3.80)	*t*(163.91) = −6.38, *p* < .001
Median	19	23	
Range	0–28	10–28	
Kurtosis	0.13	0.66	
Skewness	−0.81	−0.92	
IQR (Q1-Q3)	14.5–22	20–25	
Accuracy – Physical [Possible range, 0–14]			
Mean (SD)	8.80 (3.08)	11.88 (1.70)	*t*(177.95) = −9.00, *p* < .001
Median	9	12	
Range	0–14	7–14	
Kurtosis	−0.01	0.55	
Skewness	−0.61	−0.97	
IQR (Q1-Q3)	7–11	11–13	
Accuracy – Mental [Possible range, 0–14]			
Mean (SD)	8.87 (3.90)	10.39 (2.61)	*t*(152.55) = −3.18, *p* = .002
Median	10	11	
Range	0–14	3–14	
Kurtosis	−0.56	−0.29	
Skewness	−0.68	−0.64	
IQR (Q1–Q3)	6–12	9–12	

**Note.** Comparison *t*-tests were conducted using Welch's correction (i.e., unequal variances *t*-test) to account for heterogeneity of variances between samples.

### 3.3 Internal consistency and factorial structure

**Fig. 2:**
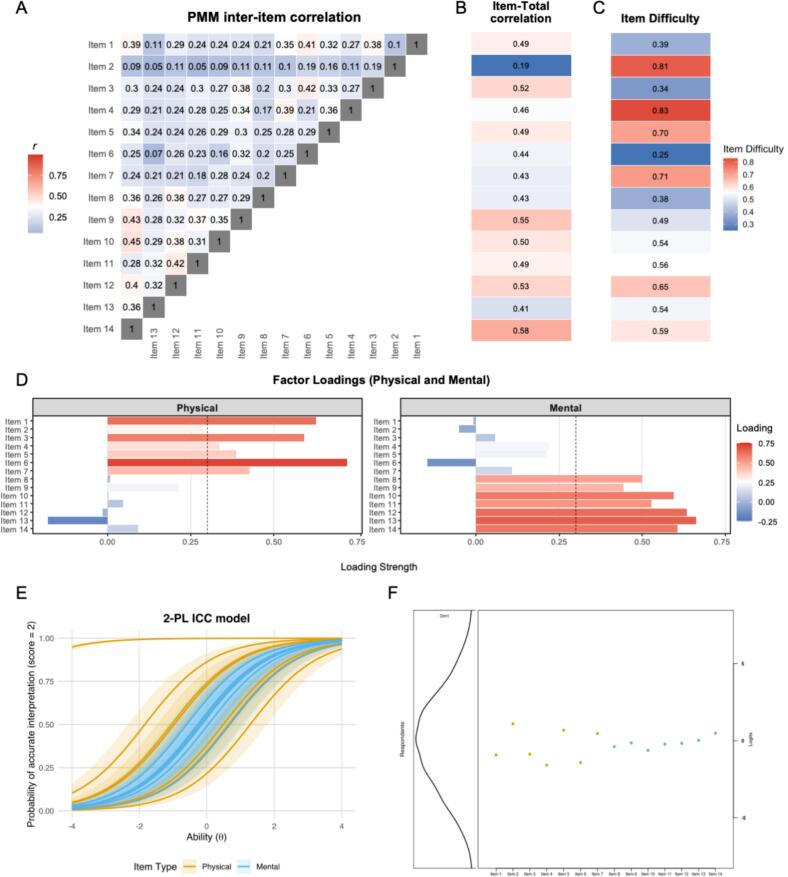
The upper part of the figure reports the Item-level psychometric properties. Panels A, B, C depict inter-item correlations and item-total correlations, and item difficulty indices (expressed as proportions, representing the percentage of participants interpreting metaphors correctly), respectively. Panels A and B share the same legend, reporting correlation coefficients (*r*) and corresponding color intensity, whereas Panel C uses a separate legend indicating the item difficulty scale. In the central part of the figure, panel D displays the results of the exploratory factor analysis, with factor loadings displayed separately for each identified factor (i.e., type of metaphors). Color intensity represents the strength of the associations, with darker shades of red indicating stronger positive correlations, blue indicating weaker (or negative) relationships, and white denoting average correlation values per analysis. In the bottom part of the figure, panel E reports the two-parameter logistic (2PL) analysis evaluating item discrimination and difficulty parameters, while panel F displays the Wright map visualizing the alignment between item difficulty and respondents' ability levels. In panels E and F, physical metaphors are shown in orange, while mental ones are shown in blue.

Internal consistency of the PMM task on the whole sample (patients and controls) indicated satisfactory reliability (*α* = 0.83, 95% CI [0.80, 0.87]; *ω* = 0.85). Average inter-item correlation was 0.26 (SD = 0.09, range [0.05–0.45]; see Fig. 2A). Corrected item-total correlations were ≥ 0.19 (M = 0.47, SD = 0.09, range [0.19–0.58]; see Fig. 2B). Item difficulty (proportion of correct answers) spanned from 0.25 to 0.83 (M = 0.55, SD = 0.18; see Fig. 2C), confirming the inclusion of metaphors with different levels of difficulty.

Exploratory factor analysis using principal-axis factoring and *promax* rotation showed the presence of a robust overarching factor (ω hierarchical = 0.65) that accounts for the largest share of variance, suggesting that metaphor comprehension is fundamentally unidimensional. At the same time, a two-factor structure also emerged, reflecting the theoretical distinction between the two types of metaphors used in the task (see Fig. 2D–F). The two-factor solution accounted for approximately 30.7% of the total variance (F1: 17.3%; F2: 13.4%). F1 showed substantial loadings for mental metaphors (≥0.43, M = 0.57, SD = 0.08, with low cross-loadings < |0.22|) and F2 acceptable loadings for physical ones (≥0.29, M = 0.48, SD = 0.16, with low cross-loadings < |0.22|). Both physical (*α* = 0.72, 95% CI [0.67, 0.78]; *ω* = 0.78) and mental metaphors (*α* = 0.78, 95% CI [0.74, 0.83]; *ω* = 0.85) showed good and acceptable internal consistency.

Item discrimination parameters ranged from 0.21 to 2.18 (M = 1.18, SD = 0.45), indicating an overall good ability to differentiate between respondents across levels of the latent trait (see Fig. 2E). Difficulty parameters ranged from −6.93 to 1.29 (M = −0.65, SD = 1.98), suggesting that the items covered a broad range of the metaphor comprehension ability continuum (see Fig. 2F).

**Fig. 3:**
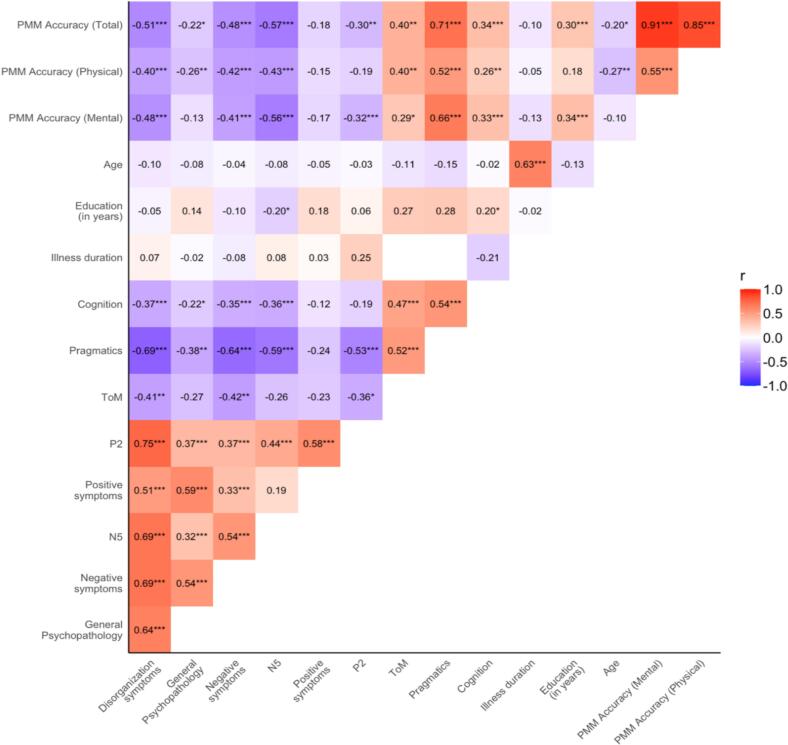
Correlation matrix between pragmatics, demographic, clinical, and cognitive variables. The heatmap illustrates the strength and direction of associations among all measured variables, with darker red shades indicating stronger positive correlations, blue shades representing negative correlations, and white indicating weak or average relationships. Statistical significance is denoted by asterisks (*p* < .05 *, *p* < .01 **, *p* < .001 ***), corrected for multiple comparisons using the false discovery rate (FDR) method.

Finally, performance in each metaphor type was also very strongly correlated with the total score (physical: *r* = 0.86, 95% CI [0.82, 0.89], *p* < .001; mental: *r* = 0.90, 95% CI [0.87, 0.93], *p* < .001), the two being moderately related (*r* = 0.56, 95% CI [0.46, 0.65], *p* < .001). See Fig. 3 for the correlogram plotting the associations between scores.

### 3.4 Test-retest and inter-rater reliability

Test-retest reliability was confirmed by a strong association between overall performances over time (*r* = 0.88, 95% CI [0.67, 0.96], *p* < .001), and moderate-to-very strong correlations for metaphor types (physical: *r* = 0.72, 95% CI [0.30, 0.90], *p* = .004; mental: *r* = 0.90, 95% CI [0.71, 0.97], *p* < .001).

The inter-rater agreement was good (ICC = 0.82, 95% CI [0.64, 0.90], *p* < .001).

### 3.5 Validity

The overview of the relationships between the PMM and pragmatic, cognitive, and clinical measures in the sample of patients with schizophrenia is provided in Fig. 3.

The test showed the expected negative correlation with age and positive correlation with education (*r* = −0.20, 95% CI [−0.36, −0.04], *p* = .031; *r* = 0.30, 95% CI [0.15, 0.45], *p* = .001). Physical metaphors accuracy scores were negatively correlated with age (*r* = −0.27, 95% CI [−0.42, −0.11], *p* = .002) but not significantly with education (*r* = 0.18, 95% CI [0.01, 0.33], *p* = .061), while mental metaphors accuracy scores were positively correlated with schooling years (*r* = 0.34, 95% CI [0.19, 0.48], *p* = .001) but not with age (*r* = −0.10, 95% CI [−0.26, 0.06], *p* = .298).^3^

#### 3.5.1 Concurrent and construct validity

Concurrent validity was assessed in the sample of patients with schizophrenia by examining the associations between PMM performance and the APACS total score. The correlation between overall PMM accuracy and APACS was strong (*r* = 0.71, 95% CI [0.56, 0.82], *p* < .001). Physical metaphor comprehension was moderately associated with APACS scores (*r* = 0.52, 95% CI [0.31, 0.69], *p* < .001), while mental metaphor comprehension was strongly associated with the performance in the APACS test (*r* = 0.66, 95% CI [0.49, 0.79], *p* < .001).

Construct validity in the sample of patients with schizophrenia was first examined by examining correlations with the cognitive abilities, namely between PMM performance and the average equivalent score from the BACS and the average score in the PST. Overall, associations were significant although weak for the PMM total scores (BACS: *r* = 0.34, 95% CI [0.18, 0.49], *p* < .001; PST: *r* = 0.40, 95% CI [0.16, 0.60], *p* = .004), physical metaphors (BACS: *r* = 0.26, 95% CI [0.09, 0.42], *p* = .007; PST: *r* = 0.40, 95% CI [0.16, 0.60], *p* = .004), and mental metaphors accuracy (BACS: *r* = 0.33, 95% CI [0.16, 0.48], *p* = .001; PST: *r* = 0.29, 95% CI [0.04, 0.41], *p* = .043).

We also evaluated concurrent validity via correlations between the PMM performance and the clinical symptom severity (PANSS items P2 and N5 and PANSS scales). Overall, PMM accuracy scores showed significant weak negative correlations with the P2 item of the PANSS (total: *r* = −0.30, 95% CI [−0.46, −0.13], *p* = .002; mental: *r* = −0.32, 95% CI [−0.47, −0.15], *p* = .001; and approached significance for physical: *r* = −0.19, 95% CI [−0.36, −0.02], *p* = .056), but not with the positive symptoms scale (total: *r* = −0.18, 95% CI [−0.35, 0.00], *p* = .068; physical: *r* = −0.15, 95% CI [−0.32, 0.03], *p* = .161; mental: *r* = −0.17, 95% CI [−0.34, 0.01], *p* = .090). N5 item, the negative symptoms and the disorganization scales were all moderately negatively correlated with PMM performance (N5: total: *r* = −0.57, 95% CI [−0.68, −0.44], *p* < .001; physical: *r* = −0.43, 95% CI [−0.57, −0.27], *p* < .001; mental: *r* = −0.56, 95% CI [−0.67, −0.42], *p* < .001; Negative scale: total: *r* = −0.48, 95% CI [−0.60, −0.33], *p* < .001; physical: *r* = −0.42, 95% CI [−0.56, −0.27], *p* < .001; mental: *r* = −0.41, 95% CI [−0.55, −0.25], *p* < .001; Disorganization scale: total: *r* = −0.51, 95% CI [−0.63, −0.37], *p* < .001; physical: *r* = −0.40, 95% CI [−0.54, −0.24], *p* < .001; mental: *r* = −0.48, 95% CI [−0.61, −0.33], *p* < .001). Finally, General Psychopathology symptoms were related to PMM total and physical metaphors accuracy scores (*r* = −0.22, 95% CI [−0.38, −0.04], *p* = .031 and *r* = −0.26, 95% CI [−0.42, −0.09], *p* = .007, respectively), but not with mental ones (*r* = −0.13, 95% CI [−0.30, 0.05], *p* = .208).

#### 3.5.2 Discriminant validity

Discriminant validity was examined by assessing the ability of the PMM task to distinguish between individuals with vs. without a pragmatic deficit (in the pooled sample including patients and controls) and between patients vs. controls.

**Fig. 4:**
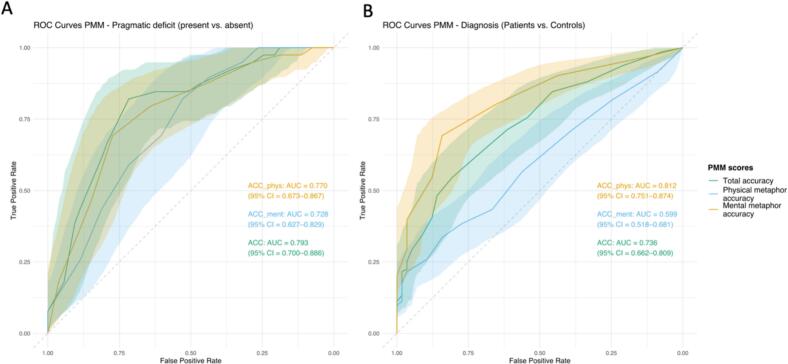
ROC curves depicting the performance in the diagnostic classification and pragmatic deficit prediction. Panel A reports the ROC for pragmatic deficit prediction. Panel B reports the ROC for diagnostic accuracy. Colors represent the three types of metaphors: scores for physical metaphors are shown in orange, for mental metaphors in blue, and for the total score in green. Ribbons display the 95% CI of each curve.

With respect to the classification of pragmatic deficit, all three PMM measures demonstrated acceptable classification accuracy, with AUC values indicating good sensitivity in discriminating between pragmatic profiles (see Fig. 4A). Specifically, PMM total accuracy score yielded the highest discrimination (*AUC* = 0.793, 95% CI [0.700, 0.866]; optimal threshold = 20.5; sensitivity = 0.82; specificity = 0.72), followed by physical (*AUC* = 0.770, 95% CI [0.673, 0.867]; optimal threshold = 11.5; sensitivity = 0.69; specificity = 0.77) and mental accuracy scores (*AUC* = 0.728, 95% CI [0.627, 0.829]; optimal threshold = 8.5; sensitivity = 0.82; specificity = 0.53). The difference in classification accuracy between Mental Accuracy and Total Accuracy was significant (*Z* = 2.22, *p* = .027), whereas the difference between Mental Accuracy and Physical Accuracy was not significant (*Z* = 0.76, *p* = .446). Similarly, the difference between Total Accuracy and Physical Accuracy was non-significant (*Z* = 0.73, *p* = .466). In sum, total accuracy demonstrated high sensitivity (hence, few false negatives) in detecting pragmatic impairments while maintaining acceptable specificity (hence, a limited rate of false positives), as much as physical metaphors, whereas mental accuracy showed lower sensitivity and specificity.

Moving to the diagnosis classification, all three PMM measures demonstrated satisfactory classification accuracy, with AUC values indicating fair-to-strong discrimination between groups (see Fig. 4B). Specifically, physical metaphor accuracy demonstrated the strongest discriminative power (*AUC* = 0.812, 95% CI [0.751, 0.874]; optimal threshold = 10.5; sensitivity = 0.69; specificity = 0.84), outperforming both total accuracy (*AUC* = 0.736, 95% CI [0.662, 0.809]; optimal threshold = 19.5; sensitivity = 0.55; specificity = 0.81) and mental accuracy scores (*AUC* = 0.599, 95% CI [0.518, 0.680]; optimal threshold = 10.5; sensitivity = 0.57; specificity = 0.56). The differences between classification accuracy values were all significant (physical vs. total scores: *Z* = 3.21, *p* = .001; physical vs. mental scores: *Z* = 5.32, *p* < .001; total vs. mental scores: *Z* = 6.68, *p* < .001). In brief, this pattern indicates moderate sensitivity (hence some false negatives) and high specificity (hence a minimal incidence of false positives) for physical metaphors, highlighting its effectiveness in correctly identifying patients and controls.

## 4 Discussion

Starting from the long-recognized, clinically informative but often overlooked difficulties with metaphor in psychosis (Bambini et al., 2020a; Mossaheb et al., 2014; Perlini et al., 2018; Rossetti et al., 2018), we addressed a gap in available neuropsychological tools by examining the reliability and validity of the Physical and Mental Metaphors (PMM) task in a sample of patients with schizophrenia. The outcome of the study offers robust evidence of the psychometric soundness and clinical utility of the PMM task as a measure of metaphor comprehension, as part of the broader pragmatic competence, and highlights its meaningful links with the psychopathological dimension and potential value as a diagnostic support tool.

With respect to the psychometric properties, the test exhibited satisfactory internal consistency and a clear factorial structure consistent with our theoretical assumptions. In particular, metaphor comprehension appears to be a unified construct, with physical and mental metaphors forming two distinct but related factors, sharing common underlying processes alongside unique characteristics based on the concrete vs. abstract properties of their implicated meanings. This factorial pattern not only corroborates theoretical distinctions between metaphor types (Winner et al., 1976) and accounts arguing for metaphor multimodality (Battaglini et al., 2025), but also reflects a fundamental unity in the cognitive mechanisms involved, aligning with prior findings in the healthy aging population (Ceccato et al., 2025). The other reliability indices further confirmed the robustness of the PMM task. Firstly, the inter-item correlation values indicate a moderate level of consistency among the items (Piedmont, 2014), implying that while the items are generally interconnected, they are not redundant. Secondly, all item-total correlation values exceed the recommended thresholds (i.e., above 0.15, see Clark and Watson, 1995), demonstrating that each item contributes meaningfully to the overall PMM score. Moreover, the item difficulty values indicate that the PMM task encompasses metaphors covering a wide range of challenge levels (Embretson and Reise, 2013), effectively allowing for the discrimination of varying degrees of metaphor comprehension ability among the patients with schizophrenia. Finally, inter-rater reliability was good, indicating that the scoring procedure can be applied consistently across raters, also of different professional backgrounds.

Crucially, the PMM task proved to be a robust indicator of the pragmatic difficulties characteristic of schizophrenia. Various results support this conclusion. First, patients performed significantly worse than controls in metaphor comprehension across all PMM scores (total, physical, and mental metaphors), reflecting the task's capability to describe a core impairment in figurative language understanding. Second, performance in the PMM strongly correlated with the performance in the APACS test, a standardized tool often used to assess the concurrent validity of tests evaluating general pragmatic skills cross-culturally and cross-diagnostically (Bischetti et al., 2025; Bischetti et al., 2024a; Bischetti et al., 2024b; Frau et al., 2025; Fussman and Mashal, 2022; Petit et al., 2025). The ROC curve analyses reinforced the link between PMM and APACS, showing also the task's good discriminative power. In particular, the PMM (both total accuracy and physical metaphor accuracy) showed good sensitivity and specificity in differentiating individuals (both patients and controls) with pragmatic deficits from those without. This series of findings has a twofold relevance. On the one hand, it supports the validity of the PMM, both concurrent and discriminant. On the other hand, the results highlight the utility of the PMM, which not only appears to be a robust tool for assessing figurative language comprehension but may also serve as a practical alternative to longer and more demanding evaluations of global pragmatic skills, to obtain patients' communicative profile.

Moving to the dimension of psychopathology, our findings confirm previous insights into the interplay between metaphor understanding and symptoms in schizophrenia (Bambini et al., 2020a; Karabanowicz et al., 2020; Mashal et al., 2013; Siddi et al., 2016). Specifically, PMM scores were negatively correlated with negative symptoms, particularly with the PANSS N5 item (i.e., “Difficulty in abstract thinking”), and with the broader disorganization symptom scale. This pattern indicates that (reduced) metaphor comprehension may represent an interface dimension between pragmatic impairment, concretism and disorganization, reinforcing the view of the tight relationship between language and thought (Abboud et al., 2026; Berrios, 1999; Palaniyappan et al., 2026; Rodriguez-Ferrera et al., 2001). While metaphor comprehension cannot be equated to difficulty with abstract thinking or disorganization, it certainly relates to these key symptoms (Bambini et al., 2020a) and reflects a relevant dimension of Formal Thought Disorder (Palaniyappan et al., 2023). These results also add novel insights into the still-debated interplay between impairment in pragmatic communication and psychopathology, suggesting that different types of figurative expressions may be differently related to symptoms. For instance, metaphors have been most frequently associated with negative symptoms and Formal Thought Disorder, whereas idiom comprehension deficits seem more closely related to positive symptoms (Sela et al., 2015) and humor is more linked to social cognition than psychopathology (Polimeni et al., 2010, but see Agostoni et al., 2024). Still, some studies failed to report an association between metaphor comprehension and symptoms (Adamczyk et al., 2024). These negative findings, while supporting that figurative language impairment does not fully overlap with negative and disorganized symptoms, may also be due to differences in the stage and clinical severity of the samples (see also Delvecchio et al., 2019 for a similar discussion on grammatical errors), as well as on the instruments used to assess metaphor comprehension, often derived from neurological scales which may fail to capture subtle nuances of concretism (such as, for instance, the Right Hemisphere Language Battery in Karabanowicz et al., 2020).

The good classification accuracy of the PMM in distinguishing patients and controls can be interpreted in light of the tight relationship between metaphor scores and symptoms, as the combination of linguistic and psychopathological aspects captured by the PMM might resemble elements used for clinical diagnosis. Interestingly, classification accuracy in distinguishing patients from controls was considerable for the physical metaphor subscale (but poor for the mental subscale). This evidence is consistent with our findings showing that physical metaphors were easier than mental metaphors for controls, whereas patients with schizophrenia did not show a comparable advantage. Hence, performance on physical metaphors seems able to provide a more precise and reliable distinction between groups (compared to the more generally difficult mental metaphors), thereby enhancing the classifier's ability to discriminate patients from controls. Furthermore, the specific pattern observed for physical metaphors might point to difficulties in schizophrenia in integrating sensory-motor properties of metaphors (Bambini et al., 2025; Mashal et al., 2014), consistent with evidence of impaired multisensory integration in psychosis (Tseng et al., 2015).

From a more general perspective, the significant, albeit modest, associations between PMM scores and general cognitive functioning and ToM skills are consistent with an extensive literature on the substrates of pragmatic abilities (Bischetti et al., 2024a; Forbes Schieche et al., 2025; Frau et al., 2024; Tomasello et al., 2025). On the one hand, metaphor comprehension relies on executive aspects such as working memory and social cognition. On the other hand, the understanding of metaphors is not wholly reducible to broader cognitive and socio-cognitive abilities (Bambini et al., 2016; Bosco et al., 2018; Bosia et al., 2016). This emphasizes the task's specificity in capturing pragmatic deficits rather than a more general cognitive impairment.

First, taken together, these findings underscore the PMM task's solid psychometric properties, as well as its clinical utility at different levels. Although several tools exist to assess schizophrenia-related traits based also on assessment of figurative language understanding (Kay et al., 1987; Kircher et al., 2014), they are coarse-grained from the linguistic point of view, and limited to proverbs. Furthermore, studies focusing on metaphor often use tools adapted from neuropsychological batteries (Chakrabarty et al., 2023; Champagne-Lavau and Stip, 2010; Karabanowicz et al., 2020; Pawełczyk et al., 2017) or designed for research purposes (Mossaheb et al., 2014; Rapp et al., 2018). In this context, the PMM task offers a novel, fine-grained instrument to describe specific difficulties with metaphors, which is known to be a vulnerable dimension with a relevant impact on functioning (Adamczyk et al., 2016; Rossetti et al., 2018). The strong test-retest stability and the high inter-rater agreement further support the reliability of the PMM, although the test-retest estimates should be interpreted with caution, given the relatively small sample available for the analysis. Moreover, its three parallel forms also make it well-suited for repeated use, enabling reliable monitoring of change over time, both during disease progression or following specific interventions.

Second, beyond metaphors, the PMM stands out as a brief yet robust assessment of pragmatic skills more globally: by targeting metaphors, PMM is able to capture a dimension that bridges deficits at the highest level of language and communication skills with clinically relevant symptoms. The capacity of the PMM to detect the presence of pragmatic deficits (as well as diagnostic status) might be particularly relevant for the screening of individuals at heightened risk, such as first-degree relatives or clinically high-risk populations, where early pragmatic or communicative alterations may precede overt symptomatology. Finally, the utility of the PMM task may extend beyond psychiatric conditions, with potential usefulness as a rapid screening tool in other populations where pragmatic difficulties are suspected, such as neurological patients or neurodevelopmental groups (Arcara et al., 2020; Bambini et al., 2020b; Cappelli et al., 2018). In a broader perspective, because metaphors permeate everyday communication, from casual conversations to the expression of emotions, intentions, and new and abstract concepts, difficulties in understanding them can significantly affect social functioning and quality of life. By capturing this subtle yet essential aspect of communication, the PMM task may provide clinicians, speech therapists, practitioners, and researchers with a window onto real-world communicative competence, offering insights that more structural linguistic measures often overlook.

## Glossary

PMM task: Physical and Mental Metaphors task
APACS: Assessment of Pragmatic Abilities and Cognitive Substrates
BACS: Brief Assessment of Cognition in Schizophrenia
PANSS: Positive and Negative Syndrome Scale
PST: Picture Sequencing Task
ROC: Receiver Operating Characteristic
AUC: Area Under the Curve

## CRediT authorship contribution statement

**Luca Bischetti:** Writing – original draft, Visualization, Methodology, Formal analysis, Conceptualization. **Valentina Bambini:** Writing – original draft, Supervision, Methodology, Funding acquisition, Conceptualization. **Giulia Agostoni:** Investigation, Data curation. **Margherita Bechi:** Investigation. **Mariachiara Buonocore:** Investigation. **Jacopo Sapienza:** Investigation, Data curation. **Federico Frau:** Investigation. **Ginevra Martinelli:** Investigation. **Chiara Pompei:** Investigation. **Biagio Scalingi:** Investigation. **Marco Spangaro:** Investigation. **Francesca Martini:** Investigation. **Federica Cocchi:** Investigation. **Roberto Cavallaro:** Resources. **Marta Bosia:** Writing – review & editing, Supervision, Project administration, Conceptualization.

## Declaration of Generative AI and AI-assisted technologies in the writing process

In the preparation of this work, the authors used GPT-5.2 and Grammarly in order to: Grammar and spelling check. After using these tools, the authors reviewed and edited the content as needed and take full responsibility for the publication's content.

## Funding

MB received support from the Italian Ministry of Research under the PRIN 2022 program, ‘The Fragility of Metaphors (FraMe): learning, losing, and how to train them’ (project number: 2022289RNA). VB received support from the 10.13039/501100000781European Research Council under the EU's Horizon Europe program, ERC Consolidator Grant ‘PROcessing MEtaphors: Neurochronometry, Acquisition and Decay, PROMENADE’ (101045733). The content of this article is the sole responsibility of the authors. The European Commission or its services cannot be held responsible for any use that may be made of the information it contains.

## Declaration of competing interest

The authors declare that they have no known competing financial interests or personal relationships that could have appeared to influence the work reported in this paper.

## Data Availability

Data of the PMM task and performance in the sample of patients and controls are available at https://doi.org/10.5281/zenodo.181200677

## Footnotes

3 Given the wider age range in the clinical group relative to controls, we ran a linear regression model testing whether age contributed differentially to PMM performance across groups. The age effect on PMM performance did not differ significantly between patients and healthy controls for any accuracy measure (overall accuracy: *b* = −0.05, *p* = .469; physical metaphor accuracy: *b* = −0.04, *p* = .235; mental metaphor accuracy: *b* = −0.01, *p* = .820), indicating that the effect of age on PMM performance is comparable in clinical and non-clinical groups.
